# Germination and Growth Analysis of *Streptomyces lividans* at the Single-Cell Level Under Varying Medium Compositions

**DOI:** 10.3389/fmicb.2018.02680

**Published:** 2018-11-22

**Authors:** Joachim Koepff, Christian Carsten Sachs, Wolfgang Wiechert, Dietrich Kohlheyer, Katharina Nöh, Marco Oldiges, Alexander Grünberger

**Affiliations:** ^1^Institute of Bio- and Geosciences, IBG-1: Biotechnology, Forschungszentrum Jülich GmbH, Jülich, Germany; ^2^Computational Systems Biotechnology, RWTH Aachen University – Aachener Verfahrenstechnik, Aachen, Germany; ^3^Microscale Bioengineering, RWTH Aachen University – Aachener Verfahrenstechnik, Aachen, Germany; ^4^Institute of Biotechnology, RWTH Aachen University, Aachen, Germany; ^5^Multiscale Bioengineering, Bielefeld University, Bielefeld, Germany

**Keywords:** *Streptomyces lividans*, filamentous growth, microfluidics, single-cell cultivation, germination, tip elongation rate, heterogeneity

## Abstract

Quantitative single-cell cultivation has provided fundamental contributions to our understanding of heterogeneity among industrially used microorganisms. Filamentous growing *Streptomyces* species are emerging platform organisms for industrial production processes, but their exploitation is still limited due to often reported high batch-to-batch variations and unexpected growth and production differences. Population heterogeneity is suspected to be one responsible factor, which is so far not systematically investigated at the single-cell level. Novel microfluidic single-cell cultivation devices offer promising solutions to investigate these phenomena. In this study, we investigated the germination and growth behavior of *Streptomyces lividans* TK24 under varying medium compositions on different complexity levels (i.e., mycelial growth, hyphal growth and tip elongation) on single-cell level. Our analysis reveals a remarkable stability within growth and germination of spores and early mycelium development when exposed to constant and defined environments. We show that spores undergo long metabolic adaptation processes of up to > 30 h to adjust to new medium conditions, rather than using a “persister” strategy as a possibility to cope with rapidly changing environments. Due to this uniform behavior, we conclude that *S. lividans* can be cultivated quite robustly under constant environmental conditions as provided by microfluidic cultivation approaches. Failure and non-reproducible cultivations are thus most likely to be found in less controllable larger-scale cultivation workflows and as a result of environmental gradients within large-scale cultivations.

## Introduction

*Streptomyces* is one of the most promising genera in industrial research for novel target molecules and structures (Bérdy, 2005; Olano et al., 2009), as their secondary metabolism facilitates the formation of more than 7,500 biologically active compounds (Bérdy, 2005). Among those are, for example the majority of antibiotics in clinical and agricultural use today (Chater, 2016). On top of that, several members of this genus, such as *Streptomyces lividans*, have been promoted as promising production hosts for heterologous proteins, due to their high secretional capacity (Sevillano et al., 2016; Anné et al., 2017). Together with the sequenced genome (Rückert et al., 2015) and established state-of-the-art genome editing techniques available (Cobb et al., 2015; Rebets et al., 2016; Wang et al., 2016), Streptomycetes are nowadays well accessible for targeted metabolic engineering approaches.

However, their fungus-like filamentous growth behavior often impedes the implementation of novel production processes (Claessen et al., 2014; van Dissel et al., 2015; Chater, 2016). High batch-to-batch variations and unexpected growth and production differences are reported frequently, caused by the complex mechanism of growth (van Wezel et al., 2006; van Dissel et al., 2014) and the linked implications on production yield and titers (D’Huys et al., 2011). So far it is known that, besides the genetic background, the apparent growth morphology (van Dissel et al., 2015) is highly sensitive at least toward media composition (Reichl et al., 1992), dissolved oxygen concentration (van Dissel et al., 2014), surface tension (Walisko et al., 2015), and energy input (Belmar-Beiny and Thomas, 1991; Koepff et al., 2017). Even the role of germling agglomeration originating from different spores, aggregating in early growth states and thereby contributing significantly to pellet size variations and culture inhomogeneity has been discussed (Zacchetti et al., 2016).

With all these variability factors in mind, the question remains how the organisms’ intrinsic heterogeneity contributes to the high batch-to-batch variations. Pinpointing cellular heterogeneity at population, spore and mycelium level may help to assess reproducibility issues and reduce the burden of unpredictability in bioprocess development. Therefore, the impact of intrinsic cellular heterogeneity as well as the contribution of different substrate complexity (here: availability of different amino-acids and complex compounds) during germination and early mycelium formation needs to be investigated.

Microscopic time-lapse imaging technologies have developed over the last three decades as preferred technique for in-depth mycelial growth investigations. The first video based analysis on the germination and mycelium formation of *Streptomyces* was already published in 1990 (Reichl et al., 1990a,b). Reichl et al., analyzed various growth parameters, such as individual hyphal length, number of branches as well as HGU. Later, the same analytical approach was applied to investigate filamentous fungi (Spohr et al., 1998; Pollack et al., 2008). Thereby, a temperature controlled growth chamber placed under a camera-equipped light microscope was used for cultivation. In further time-lapse studies *Streptomyces* was grown on solid media, either using small agar patches (Hempel et al., 2008) or plugs (Jyothikumar et al., 2008; Willemse et al., 2012), flattening the three-dimensional mycelial structures. Recently, a microfluidic cultivation device was applied to investigate the whole *Streptomyces* life-cycle, including germination, mycelium formation and sporulation (Schlimpert et al., 2016). Nowadays, such miniaturized cultivation systems can be operated at highly constant environmental conditions regarding the cultivation medium, temperature and oxygen concentration. At the same time single microfluidic devices enable high throughput analysis and the application of statistical methods due to a high degree of parallelization (Dusny and Schmid, 2015). These unique features allow investigating large cell numbers to unravel cell-to-cell heterogeneity induced by biological and intrinsic factors, independently from environmental fluctuations. Xu and Vetsigian (2017) investigated spores of *Streptomyces* disclosing phenotypic variability and interactions between communities. Performing cultivations on agarose-pads, they systematically investigated the sporulation and growth of four different *Streptomyces* strains and identified differences in the germination fraction, depending on the strain and differences in the level of medium dilution.

In this study, we investigated the growth characteristics as well as the variability in germination and early stage mycelium development of *S. lividans* TK24 via MSCC inside hundreds of picoliter sized growth chambers. Live-cell imaging and subsequent data analysis was performed by a tailor-made image based data processing workflow. In a first step, our new MSCC approach was validated by accompanying MTPC applied as our reference system. In a second step, six different media compositions with decreasing complexity in terms of available substrate spectrum were applied to investigate phenotypic growth variations of *S. lividans* on the level of entire populations, single spores, and individual mycelia.

## Materials and Methods

### Strains and Media

All experiments were conducted using *S.* *lividans* TK24, a plasmid-free derivative of *S.* *lividans* 66 (Cruz-Morales et al., 2013), which is a close relative of *S.* *coelicolor* A3(2), generally regarded as the best genetically characterized *Streptomyces* strain (Hopwood, 1999).

To prepare *S. lividans* spore solutions the protocol published by Kieser et al. (2000) was applied: Solid corn steep agar (Hobbs et al., 1989) cultures were grown for 10 days at 28°C to obtain well-sporulated mycelium. By pipetting 10 mL sterile PBS with 20% (vol/vol) glycerol onto an agar surface and gentle scratching with a sterilized spreader rod, the spores were dispersed and subsequently separated form remaining hyphae fragments by a custom-made cotton wool filter, before being stored at -20°C as permanent culture.

For main-culture in chip or MTP cultivations, all together six different media compositions were applied (Table 1). Full complex phage medium (CM) (Korn et al., 1978), consists of 5 g⋅L^-1^ peptone, 5 g⋅L^-1^ yeast extract, 5 g⋅L^-1^ meat extract, 0.5 g⋅L^-1^ MgSO_4_⋅7 H_2_O and 0.74 g⋅L^-1^ CaCl_2_⋅2H_2_O. The basic minimal medium was composed of 2.07 g⋅L^-1^ NaH_2_PO_4_⋅1 H_2_O, 2.6 g⋅L^-1^ K_2_HPO_4_, 0.6 g⋅L^-1^ MgSO_4_⋅7 H_2_O, 3.0 g⋅L^-1^ (NH_4_)_2_SO_4_ and 10 g⋅L^-1^ D-glucose, 100 mM 2-(N-morpholino)ethanesulfonic acid (MES) as well as several trace elements (1 mg⋅L^-1^ ZnSO_4_⋅7 H_2_O, 1 mg⋅L^-1^ FeSO_4_⋅7 H_2_O, 1 mg⋅L^-1^ MnCl_2_⋅4 H_2_O, 1 mg⋅L^-1^ CaCl_2_), having the pH adjusted to 6.8 using 1 M H_2_SO_4_. To supplement the minimal media, either 2 g⋅L^-1^ hydrolyzed milk protein (Bacto^TM^ Casamino Acids, MM_CAS) or 2 g⋅L^-1^ of 3 different amino acids mixtures were added (Table 1), with ratios following the natural abundances in *Streptomyces* protein (Kieser et al., 2000). Additionally, minimal media without any supplementation was used as reference medium. The detailed compositions are provided in Supplementary Material S1.

**Table 1:** Media compositions and supplementation for both: chip- and MTP-based cultivation.

Medium ID	Medium basis	Supplementation	Final concentration
CM	Complex phage medium	–	–
CAS	Minimal medium	Bacto^TM^ Casamino acids	2 g⋅L^-1^
AA20	Minimal medium	All 20 proteinogenic amino acids	2 g⋅L^-1^
AA08	Minimal medium	Arg, Asn, Asp, Glu, Leu, Met, Phe, Thr	2 g⋅L^-1^
AA04	Minimal medium	Asn, Leu, Met, Phe	2 g⋅L^-1^
AA00	Minimal medium	–	–

*Selection for 8 (MM_08) and 4 (MM_04) amino acids was done according to the proposal in Nowruzi et al. (2008). The individual amino acid concentrations are provided in Supplementary Material S1*.

### Microfluidic Cultivation Conditions

**FIGURE 1:**
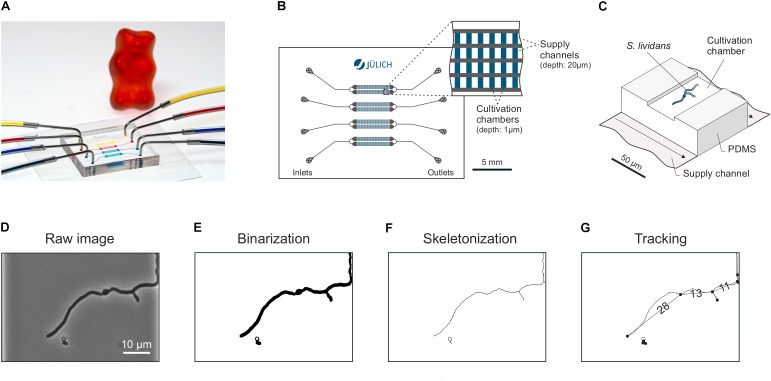
Microfluidic single-cell cultivation (MSCC) and selected steps of the image based data analysis pipeline. **(A)** Photographic representation of the PDMS chip with connected in- and outlet tubing. Colorized liquids were used for demonstration purposes. **(B)** Schematic chip geometry. Four individual fluid lines with separate in- and outlets are arranged in 8 x 40 cultivation chamber arrays. **(C)** 3D illustration of the supply channels and a single cultivation chamber. **(D–G)** Major steps of the image processing pipeline: Raw images **(D)** were binarized **(E)** before being converted into skeletons **(F)** which could be subsequently analyzed by spatiotemporal tracking **(G)** in terms of total branching length and mycelial sections (Sachs et al., 2018 (in preparation)).

Microfluidic single-cell cultivations (MSCCs) were performed using a modified version of the polydimethylsiloxane (PDMS) cultivation device described by Grünberger et al. (2015) (Figure 1A). The chip system consists of 8 arrays of monolayer cultivation chambers (l × w × h = 90 × 40 × 1 μm), with 40 chambers each (Figures 1B,C). For fabrication details the reader is referred to Grünberger et al. (2013). The microfluidic chip was mounted onto a motorized inverted microscope (Nikon Eclipse Ti, Nikon, Japan) equipped with an incubator (PeCon Series 2000, PeCon GmbH, Germany) for temperature control. A *S. lividans* spore suspension with a concentration of ∼10^6^ spores⋅mL^-1^ was inoculated into the chip, according to the loading procedure as described earlier for *Escherichia coli* (Probst et al., 2015). Fluidic connections were established by silicone tubing (Tygon S-54-HL, ID = 0.25 mm, OD = 0.76 mm, Saint-Gobain, United States) and dispensing needles (ID = 0.2 mm, OD = 0.42 mm, Nordson EFD, United States). Media were infused at approx. 200 nL⋅min^-1^ after cell inoculation by a syringe pump (neMESYS, cetoni GmbH, Germany). The media described above were systematically tested as growth media. Specific growth chambers which were most suitable for imaging were selected manually. Each experiment was stopped, as soon as the mycelia started to overgrow the chamber volume.

### Image Processing and Data Analysis

Image analyses were performed using the analysis software *mycelyso* (Sachs et al., 2018). For each image frame, the growth chamber was automatically detected and used as a region of interest (ROI), removing the need for separate image registration. Time lapse image stacks (Figure 1D) were segmented (Figure 1E), skeletonized (Figure 1F) and automatically tracked (Figure 1G) using the *mycelyso* software. Automated filtering (standard *mycelyso* settings) during the tracking process was applied in order to remove artifacts due to tracking errors: (a) tracked hyphae must be tracked for at least 5 consecutive time points, (b) they must reach a length longer 10 μm at the end of the track, (c) be grown for at least 5 μm, (d) hyphae must not have an elongation rate greater than 100 μm ⋅ h^-1^. To exclude erroneous segmentations due to densely packed mycelia, tracking was only performed until 20% coverage of the growth chamber was reached. Analysis results were visually checked using *mycelyso* Inspector, and a subset of positions was chosen, which (i) contained a properly grown mycelium (centered, viable) and (ii) were analyzed without obvious errors. Data was collected by *mycelyso* in hierarchical data format 5 (HDF5) files and further subjected to statistical evaluation using Python with commonly available modules (i.e., numpy, scipy, pandas, and matplotlib): data of individual positions was collected for interpretation and visualization, means and standard deviations calculated. Germination delay was determined for each position by the time where the first trackable hypha occurred. Growth rate of the total mycelium length was determined per chamber by assuming exponential growth and employing a log-linear regression model with *R*^2^ > 0.9. HGU was determined by finding the maximum of the quotient the smoothed time-resolved data “total mycelium length” divided by “count of tips” during the exponential growth phase. Means of different media compositions were statistically assessed using both equal and unequal variance (Student’s/Welch’s) *t*-test. Data analysis and figure generation is available as a Jupyter notebook in the Supplementary Analysis Data (Supplementary Data Sheet S2).

### Microtiter-Plate Cultivation (MTPC) of *S. lividans*

Microtiter-plate cultivation was performed in 48-well FlowerPlates© (MTP-48-BOH) covered by gas permeable sealing foils (F-GP-10) in an automated cultivation device (BioLector, m2p-labs GmbH, Germany) as previously described (Koepff et al., 2017) with the following modifications: Wells, prefilled with 1000 μL cultivation medium were inoculated using 50 μL spore suspension with an concentration of ∼10^6^ spores⋅mL^-1^. Cultivation parameters were set to 800 rpm shaking frequency, cultivation temperature of 30°C, 85% relative humidity and a 10 min recording interval for scattered light measurements. In all experiments MTP-cultivation was performed parallel to chip experiments, using the identical spore vial for inoculation, ensuring identical start conditions for all cultures.

### Data Analysis of MTPC Online Signals

Scattered light signal was blanked in each well separately, by the average of the first 10 values. Lag-phase was calculated as the time difference between cultivation start and the beginning of exponential-like growth behavior in the scattered-light signal. Specific growth rate was determined on spline interpolation (10 knot points) of scattered light data as described elsewhere (Radek et al., 2017).

## Results and Discussion

### Validating Microfluidic Single-Cell Against Submerse Microtiter-Plate Cultivation

**FIGURE 2:**
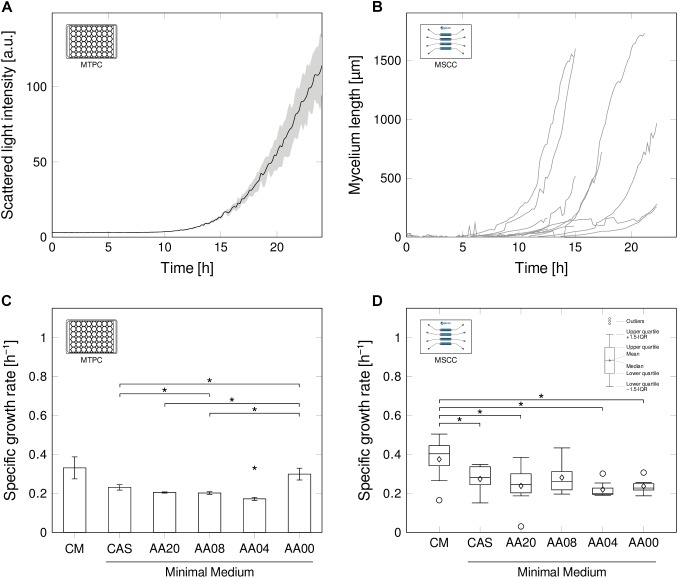
Growth behavior of *Streptomyces lividans* in MTPC **(A,C)** and MSCC **(B,D)**. **(A)** Scattered light intensity obtained from MTP cultivation (MTPC), exemplarily for CM (*n* = 3), using a spore suspension for inoculation. **(B)**: Total mycelium length [μm] over process time for *n* = 8 representative spores in microfluidic MSCC all grown in CM; image analysis was stopped when complexity of mycelium impaired evaluation. **(C,D)**, Specific growth rates for six media compositions (each *n* = 3) with decreasing level of complexity (Table 1) in the MTPC and MSCC. Significant differences (*p* < 0.05) are indicated by an asterisk (^∗^).

For research and production purposes, *S. lividans* is typically grown in agitated bioreactor environments in submerse cultures (Kieser et al., 2000; van Dissel et al., 2014). Under such conditions, the organism shows normal growth kinetics in biomass related signals, such as scattered light intensity (Figure 2A). After a lag-phase, in which no growth is detectable, an exponential increase of scattered light is recorded, then limitations occur, which cause stagnation and at a later stage even decrease of the signal pattern.

However, the optically derived scattered light signal, alike all other macroscopic biomass signals, describes an average status of the culture as it is affected by all scattering particles within the liquid volume. Its aggregate character therefore is not suitable to unravel mycelial growth in detail: It remains unclear whether the flat-line signal pattern within the first hours of cultivation reflects a biological adaption process (lag-phase) before germination of the spores, or if the growth activities are simply occurring below the signal detection limit. Therefore, such averaged signals cannot be used to resolve heterogeneities and variability occurring within the mycelium of individual spores and in-between mycelia emerging from different spores.

To disclose the activity of single spores and single mycelia, we cultivated *S. lividans* inside MSCC devices under continuous time-lapse imaging (Figure 2B). PDMS based chip manufacturing enabled us to tailor an application-specific microfluidic device (Figure 1), suitable for *S. lividans* cultivation in its early stage of germination. The height of the growth chambers (∼1 μm) in the used device approximately match the diameter of single spores thereby aligning the cellular growth in 2D monolayers. Constant environmental conditions were ensured by the continuous and balanced medium supply adjacent to both openings of each cultivation chamber.

Evaluating the total development of mycelial length for individual spores grown in CM (Figure 2B) basically revealed a comparable exponential growth pattern in comparison to the MTPC reference system. Nevertheless, the microfluidic analysis unraveled the presence of spore-to-spore variation, as the culture-wide scattered light signal indicated. Growth initiation occurred in a wide temporal distribution, but also the exponential growth behavior varied between individually evolving mycelia. Still, growth rates obtained from the MTP reference system (Figure 2C) and from single spores in the microfluidic chip (Figure 2D) approach are generally comparable under all six investigated media compositions, ranging from full complex (CM), to pure minimal medium (AA00) with supplemented minimal medium steps in-between (for detailed media compositions see Table 1). Once more, this notable result underlines, that MSCC can mirror submerse conditions (Grünberger et al., 2013).

As expected, the highest specific growth rates (0.38 h^-1^ ± 0.11 h^-1^ in MSCC, 0.33 h^-1^ ± 0.06 h^-1^ in MTPC) were obtained in CM, as a broad spectrum of available substrates facilitates the faster formation of new mycelial structures. In the remaining media compositions, the estimated growth rates ranged in MSCC between 0.27 h^-1^ ± 0.07 h^-1^ for CAS and 0.22 h^-1^ ± 0.04 h^-1^ for AA04. In MTPC, the growth rates were 0.23 h^-1^ ± 0.01 h^-1^ for CAS and 0.17 h^-1^ ± 0.01 h^-1^ for AA04. Thus the growth rate on CM was significantly higher, compared to the growth rate of the media compositions CAS, AA20, AA04, AA00 (all *p* < 0.05). No significant growth rate difference was found between CM and AA8 (*p* = 0.11).

The slightly higher growth rates, obtained in MSCC (see Supplementary Material S2) fits well to previously reported results for different biological systems and cultivation conditions (Unthan et al., 2014; Dusny and Schmid, 2015). This trend may be caused by the differences of the cultivation modes (batch vs. continuous) or, in case of MTPC, potentially the presence of nutrient gradients in the cultivation wells leading to temporally changing conditions. This renders a direct and absolute rate comparison between the two systems difficult.

Interestingly, not only the growth rate, but also growth rate distributions were found to be a function of the media complexity: Highest growth rate fluctuations between individual spores as well as between parallel wells in the MTP setup were observed in CM. These fluctuating rates may contribute to pellet size distributions, as recently reported for CM cultivations (Zacchetti et al., 2016). Also, significantly reduced size distributions were reported herein for minimal media setups, which is again an agreement to the rate fluctuations in the corresponding media setups (Figure 2D).

### Spore Germination Time Point Considering Inner-Culture Variations

**FIGURE 3:**
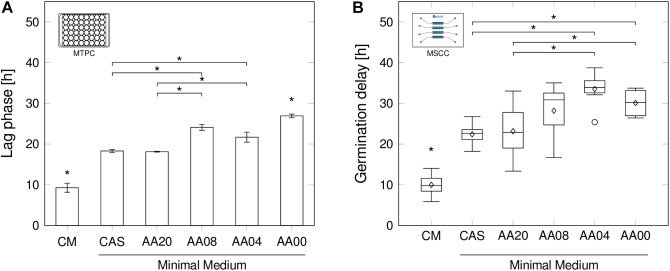
Lag-phase and germination delay by decreasing medium complexity. **(A)** Lag-phase durations in shaken MTPC for all six medium conditions. Error bars calculated from *n* = 3 biologically independent replicates. **(B)** Germination delays determined by MSCC in *n* ≥ 6 cultivation chambers. Box plot annotation identical to Figure 2D. Determination methods for lag-phases and germination delays are provided in the methods section. Significant differences (*p* < 0.05) are indicated by an asterisk (^∗^).

Whereas in MTPC and other established cultivation systems only average lag-phases are determinable, MSCC allows investigating the initial delay until germination of individual spores (Figure 3).

A clear dependency of the medium complexity on the germination delay, namely the time difference between inoculation and spore germination, was observed in both cultivation systems (Figure 3). During MSCC (Figure 3B) the spores began to form primary hyphae after already 10.0 h ± 2.4 h, when being cultivated in CM, while significant longer delays were found in all other media applied in this work (up to 33.5 h ± 3.9 h in AA04) (all *p*-values < 0.05). Interestingly, very comparable mean values were obtained for the complex amino acid combination CAS and the artificial AA20 mixture. In both media spores show a significant faster spore germination compared to medium AA04 and AA00 (all *p*-values < 0.05). In contrast, the media with 4 (AA04) and 8 (AA08) supplemented amino acids do not have a positive influence on the germination delay, since the non-supplemented negative-control (AA00) showed comparable delays.

Remarkably, the mentioned effects above, obtained by chip-based cultivation (Figure 3B), showed equivalent tendencies for the observed lag-phase durations, derived from MTPCs (Figure 3A). The absolute values ranged between 9.3 h ± 1.1 h for CM and 27.0 h ± 0.5 h in AA00 medium. Although the cultivation systems highly differ by design and mode of operation, average spore germination seems not to be affected significantly.

The lag-phase is generally understood to be an adaptation process upon a change of the environmental condition, possibly resulting in a homogenous switching into a new phenotype or a phenotypic (stochastic vs. responsive) diversification into two phenotypes (Kotte et al., 2014). In the present study, these altered conditions may include the availability of substrates. Here, the remaining question depicts the occurrence of intrinsic cellular heterogeneity toward germination time points, as it may illuminate underlying regulation mechanisms and adaptation strategies. By studying the distribution of the single-spore germination time points, we found that interestingly all germinating spores in the observed growth chambers showed quite robust and consistent behavior with standard deviations between 2.4 h (CM, 10.0 h ± 2.4 h) and 3.9 h (AA04, 33.5 h ± 3.9 h).

In opposition to that, when some spores show a rapid germination, while others remain dormant as persistent cells, backing up the population in case of emerging more disadvantageous conditions a bimodal behaviour would have been suspected. Such a bimodal behavior has been observed for microbial adaption processes upon the availability of different carbon sources (Solopova et al., 2014). Differences in the germination delay in CM and between the minimal media might be due to the modifications in amino-acid availability (Ensign, 1978) or a combination with other unknown complex medium compounds, rather than the cultivation method. This lays the foundation, to use MSCC as a future screening tool for further germination studies with different medium compositions. Please note, that the overall spore germination behavior is quite low (36.12%, Supplementary Material S3), but comparable for all media (CAS = 31%; AA20–AA00 = 33%), while only with CM higher outgrowth values (CM = 61%) were reached. The latter number is well comparable to published results, where in similar CM a germination fraction of ∼45% was observed after 24 h for *S. coelicolor* (Xu and Vetsigian, 2017). Interestingly, during the inoculation procedure of spores into separate cultivation chambers and subsequent continuous perfusion with fresh cultivation medium, the accumulation of germicidins (germination inhibiting agents) was presumably avoided. These substances were reported to be secreted by germinating spores to prevent the germination of neighboring spores (Aoki et al., 2011; Xu and Vetsigian, 2017), but should not have an influence during MSCC.

### Intra-Mycelial Variations: Hyphal Elongation Rates of Individual Apical Growth Tips – Within a Single Spore and In-Between Spores

**FIGURE 4:**
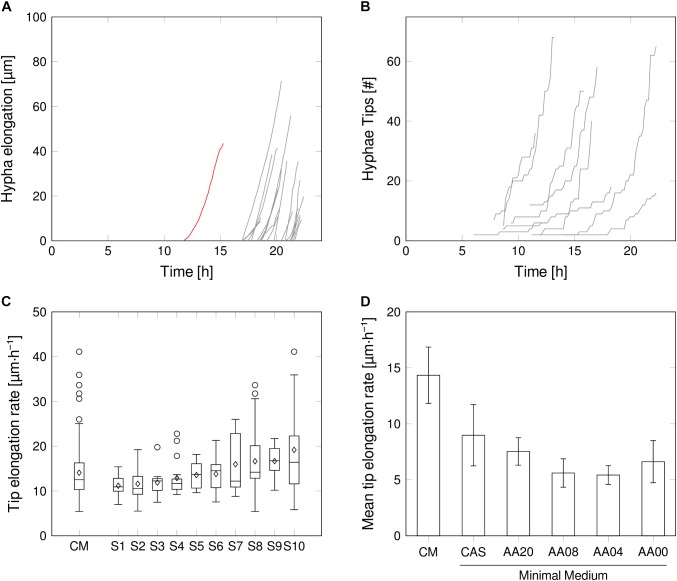
Intrinsic growth heterogeneity on mycelium level. **(A)** Additional length of the developing mycelium in one representative chamber. Primary hyphae (red) and subsequent hyphae after branching (gray). **(B)** Number of hyphal tips, exemplarily for several spores in complex medium. **(C)** Tip elongation rate distribution for all and 10 representative spores supplied with CM. Outliers may be provoked by missing or falsely annotated links between image frames. **(D)** Average tip elongation rate (average of mean tip elongation rate of all evaluated spores of each medium condition). Detailed tip elongation rates for each medium condition are provided in Supplementary Material S4.

Beside the previously discussed spore-level parameters, image analysis allows to take a deeper look at underlying inner-spore growth performances and fluctuations. This was realized by dissecting the developing mycelium into individual hyphae segments, separated at the branching points (Figures 1G, 4A). Notably, the primary hypha, arising from the spore, showed an approximate exponentially increasing tip elongation rate within the first hours after germination. In contrast, all subsequent branches grew at linear rates right after branching. A similar growth pattern discrimination between the first and subsequent mycelial segments was already previously reported (Allan and Prosser, 1983; Kretschmer, 1988; Reichl et al., 1990a; Yang et al., 1992). This underlines the suitability of the developed microfluidic cultivation system for comparative growth studies. The exponential development of the total mycelial length therefore is a result of the exponential increase of branching points rather than of the growth of the individual tips (Figure 4B).

Within the mycelial network of a single spore, the elongation rates fluctuated strongly (Figure 4C), resulting in a mean elongation rate of 14.1 μm⋅h^-1^ ± 6.1 μm⋅h^-1^ in CM, over all observed spores (Figure 4C). However, this points to a robust phenomenon, as no pronounced variations in the rate distribution were found when comparing different spores, supplied by the same medium (Figure 4D and Supplementary Material S2). Thus, inner-spore tip elongation rate variations probably will not have a strong effect on macroscopic heterogeneities. Here, the limited chamber height due to fabrication design could influence data interpretation, as it may lead to friction of hyphae and result in unsteady growth behavior over time. The same holds true for inaccuracies within cell detection over time, due to limitations of the automated image-analysis workflow.

The HGU, which describes the average length of a hyphal segment, follows a similar trend as the mean tip elongation rate (Supplementary Material S5). Highest HGU was found in CM with an average of 28.7 μm, which is in well accordance with previous results for *S. tendae* (Reichl et al., 1992) and *S. coelicolor* (Allan and Prosser, 1983) on a comparable medium. Interestingly, various amino acid supplementations did not have a strong influence on the HGU as all remaining media setups resulted in average HGUs between 14.3 μm (AA04) and 17.4 μm (CAS). Even though there is not much known about the effect of minimal media supplementation on HGUs in filamentous bacteria, in tendency reduced HGUs have been reported for MM setups in comparison to CM, but with a high dependency on carbon (glucose) availability (Reichl et al., 1992). In the present study, however, all minimal media setups contained an identical concentration of glucose (10 g⋅L^-1^) and, except for AA00, a stoichiometrically adjusted amount of amino acids. From our results such a general relation between richness of medium and branching frequency cannot be deduced. By far the strongest fluctuations in HGUs between spores were recorded for CM as expressed by a standard deviation of 21.7 μm. The larger pellet size variations reported for complex compared to minimal media (Zacchetti et al., 2016) may therefore be explained by this issue. In minimal media setups reduced HGU fluctuations were obtained where the variations decrease with the medium complexity.

## Discussion

Inner-culture variability resulting in unfavorable fluctuation in growth and production are repeatedly reported for members of the *Streptomyces* genus, and are known to constitute a risk when employing members of this genus in industrial production processes (van Dissel et al., 2014). Several genetic and especially environmental effectors have been identified over the years, known to contribute to the observed cultivation difficulties. As bulk measurements can occlude the observation of heterogeneously reacting subpopulations, we choose to investigate inner-*Streptomyces* heterogeneity using MSCC in combination with an automated image analysis, allowing for individual, unbiased investigation of growth parameters per spore and mycelium.

We validated the MSCC for filamentous *Streptomyces* against an established MTP cultivation method (Koepff et al., 2017). Here a high comparability was obtained between the two systems in germination and early-stage mycelium formation. Differing not only in working volume by a factor of 10^6^, but also by their cultivation mode (perfusion vs. batch), this was a notable finding, confirming the applicability of MSCC in further screening and in-depth growth investigations for filamentous bacteria. The MSCC has therefore proven – again – to be a useful and efficient tool to deeply investigate inner-culture heterogeneity also in filamentous organisms. To operate this technology to its potential, time-lapse microscopy accompanied by a powerful image processing and data analysis software pipeline is a necessary prerequisite. By analyzing ≈ 680 GB of image stacks in a reproducible and unbiased manner, it is possible to draw comprehensive statements across different hierarchical morphological levels that would otherwise be impossible to derive.

Even though there are variation in mycelium growth, germination and tip elongation, these variations are a reproducible phenomenon in each set of spores and therefore probably not the driving force behind discussed reproducibility issues and inner-culture heterogeneities. While CM provides a faster growth performance in all recorded parameters, it goes along with the burden of higher fluctuations on spore level (average mycelium growth rate, germination time delay) and mycelium level (tip elongation rate, HGU). When inner-culture homogeneity is the main objective, minimal medium setups are to be preferred. Even though there are inner-culture fluctuations, especially due to individual spore germination delays, still a robust inner growth performance of the organism prevails. In conclusion, this single-cell characterization shifts the focus back to the already known, especially environmental factors such as gradients within large-scale processes (Lara et al., 2006) and variabilities/variances within cultivation workflows (Koepff et al., 2017), known to trigger batch-to-batch and inner-culture fluctuations. Filamentous organisms, even more as their bisecting microbial competitors, demand for highly standardized and constant cultivation conditions. If such conditions are provided, it should be possible to obtain reproducible cultivation results. This strongly underlines the need to investigate the impact of gradients in large-scale production when using filamentous organisms, as done manifold for bisecting production hosts such as *E. coli* (Löffler et al., 2016) and *C. glutamicum* (Limberg et al., 2017).

Further progress within microfluidic fabrication techniques, will allow the investigation of more complex biological questions in MSCC, for example the emulation of large-scale bioprocesses by exposing cells to altering medium conditions over time (Binder et al., 2017). An improved understanding of the origin, reason and impact of heterogeneities in *Streptomyces* large-scale cultivation will lay the foundation for improved bioprocesses and cultivation pipelines.

## Author Contributions

JK and AG conceived the study. JK and AG performed the experiments. CS analyzed the data. JK, AG, and CS interpreted the results. JK and CS wrote the first draft. JK, CS, DK, KN, MO, and AG discussed the findings. WW, DK, KN, MO, and AG copyedited the manuscript. WW provided the funding.

## Conflict of Interest Statement

The authors declare that the research was conducted in the absence of any commercial or financial relationships that could be construed as a potential conflict of interest.

## Abbreviations

AA00: Pure minimal salt cultivation medium
AA04: Minimal salt medium, supplemented 4 selected amino acids
AA08: Minimal salt medium, supplemented 8 selected amino acids
AA20: Minimal salt medium, supplemented with all 20 proteinogenic amino acids
CAS: Minimal salt medium, supplemented with Bacto^TM^ Casamino Acids
CM: Complex cultivation medium
HGU: Hyphal growth unit
MSCC: Microfluidic single cell cultivation
MTPC: Microtiter-plate cultivation
*S. lividans*: *Streptomyces lividans* TK24
