# Supplementary material for: Germination and Growth Analysis of Streptomyces lividans at the Single-Cell Level Under Varying Medium Compositions
Source: Front Microbiol. 2018 Nov 22;9:2680. doi: 10.3389/fmicb.2018.02680 (PMC6262040; doi:10.3389/fmicb.2018.02680)
Supplement: Supplementary file 1 [file Data_Sheet_1.pdf]

Supplementary Material: Germination and Growth Analysis of *Streptomyces lividans* at the Single-Cell Level Under Varying Medium Compositions

Joachim Koepff [1†], Christian Carsten Sachs [1†], Wolfgang Wiechert [1,2], Dietrich Kohlheyer [1,3], Katharina Nöh [1], Marco Oldiges [1,4] and Alexander Grünberger[1,5,*]

[1] *Institute of Bio- and Geosciences, IBG-1: Biotechnology, Forschungszentrum Jülich GmbH, Jülich, Germany,*
[2] *Computational Systems Biotechnology, RWTH Aachen University – Aachener Verfahrenstechnik, Aachen, Germany,*
[3] *Microscale Bioengineering, RWTH Aachen University – Aachener Verfahrenstechnik, Aachen, Germany,*
[4] *Institute of Biotechnology, RWTH Aachen University, Aachen, Germany,*
[5] *Multiscale Bioengineering, Bielefeld University, Bielefeld, Germany*

[†] *These authors have contributed equally to this work*

Correspondence*:
Alexander Grünberger
alexander.gruenberger@uni-bielefeld.de

SUPPLEMENTARY TABLES AND FIGURES

S1: Amino acid composition for supplementations of minimal medium approaches

Beside Bacto™ Casamino acids, which consist of hydrolyzed milk protein, the minimal medium was supplemented with mixtures of amino acids in different complexity (Table S1). Either all 20 proteinogenic amino acids (MM_20), or reduced sets, proposed by Nowruzi et al. (2008), with 8 (MM_08) or 4 (MM_04) different components were added. The ratios of the single amino acids were calculated by the occurrence in the Streptomyces protein (Kieser et al. 2000). Additionally, a negative control without any supplementation was prepared.

Table S1. Amino acid supplementation of minimal medium

**Substance**	**Occurrence in Streptomyces protein [%]**	**Medium name, supplementation [g·L^{-1}]**			
		**MM_20**	**MM_08**	**MM_04**	**MM_00**
Ala	13.1	0.66	–	–	–
Arg	8.3	0.42	0.95	–	–
Asn	2.4	0.12	0.28	0.73	–
Asp	6.4	0.32	0.73	–	–
Cys	0.9	0.05	–	–	–
Gln	2.6	0.13	–	–	–
Glu	5.9	0.30	0.68	–	–
Gly	9.1	0.46	–	–	–
His	2.4	0.12	–	–	–
Ile	3.3	0.17	–	–	–
Leu	9.6	0.48	1.10	2.93	–
Lys	2.4	0.12	–	–	–
Met	1.6	0.08	0.18	0.49	–
Phe	2.8	0.14	0.32	0.85	–
Pro	5.5	0.28	–	–	–
Ser	5.4	0.27	–	–	–
Thr	6.6	0.33	0.76	–	–
Trp	1.5	0.08	–	–	–
Tyr	2.2	0.11	–	–	–
Val	8.1	0.41	–	–	–
**Sums**	100	5	5	5	0

S2: Growth rate: MTPC vs. MSCC

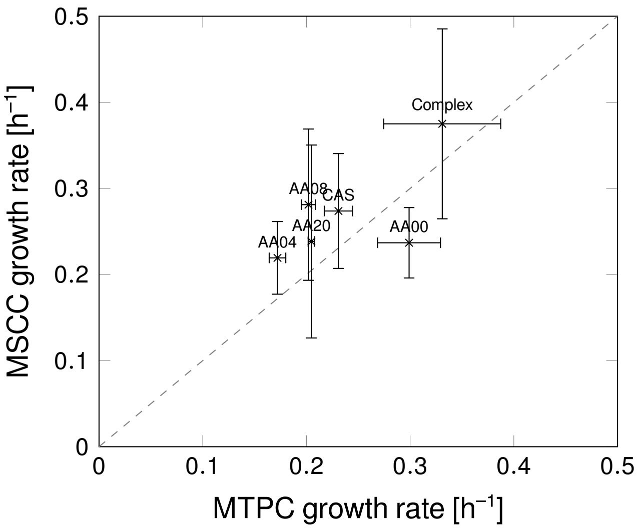

Figure S2. Growth rate determined in MTPC vs. growth rate determined in MSCC. Comparing illustration of growth rates shown in Figure 2 D-E. Error bars: standard deviation.

S3: Outgrow behavior distribution of *S. lividans* spores

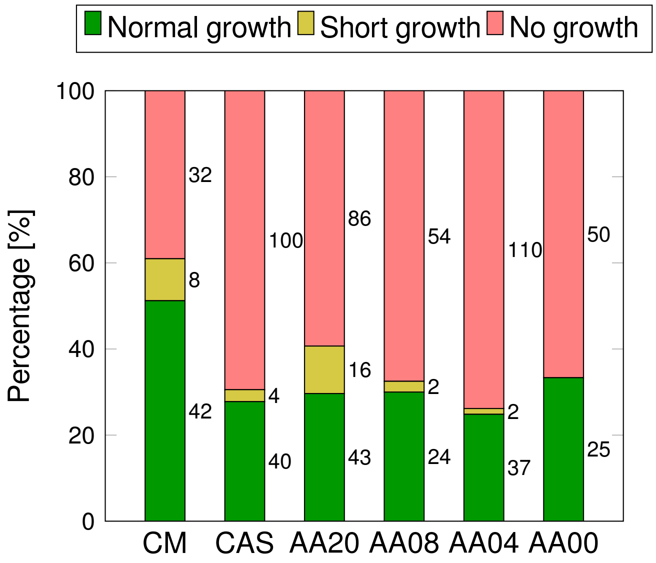

Figure S3. Outgrow behavior distribution of S. lividans spores.
Relative amounts of observed chambers with single spores in MSCC grouped in stacked bars by the respective spore outgrow behavior. Normal outgrow (green), interrupted /aborted outgrow (yellow) and no observed outgrow (red), absolute spore count (small numbers).

To facilitate image analysis, positions which showed proper, likely automatically analyzable growth behavior were picked. To assess overall viability, all positions with growth structures were manually inspected by looking at a collage of six timepoints (equitemporally spaced) and categorized into four categories depending on this overview: Normal growth (i.e. mycelium is seen growing till the end), short growth (i.e. mycelium is seen to grow for some time, but does not seem to continue growing till the end), and no growth (i.e. spore does not germinate). The distribution of these three categories for each medium composition is shown in Fig. S3. The fourth category contains unclear cases (data not shown in Fig. S3), which could not be resolved by looking at the six timepoints, mostly spores which were overgrown from mycelium entering the growth chamber from the outside, occluding whether the spore itself would germinate, and making image analysis infeasible. The unclear category is comprised of CM n=9, CAS n=10, AA20 n=23, AA08 n=4, AA04 n=4.

S4: Tip elongation rates within individual spores in different media

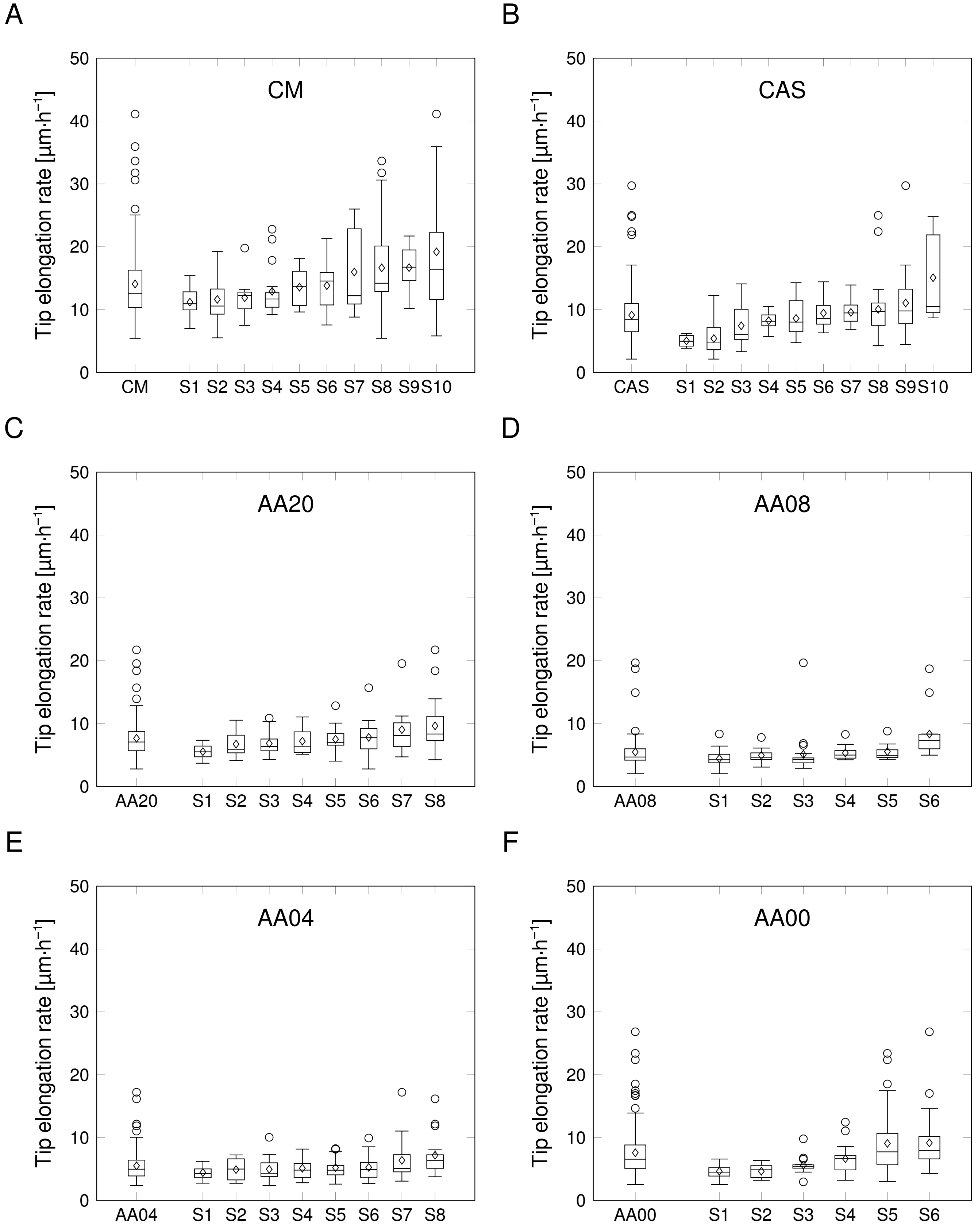

Figure S4. Tip elongation rate distribution of growth tips in exemplary spores.
Grouped by the respective cultivation medium: Complex medium (A), CAS medium (B), and media with decreasing amino acid supplementation (C-F). First box plot in each figure: tip elongation rates of all tips of the shown spores. Box plot annotation as indicated in Figure S5.

S5: Intra-mycelial variations heterogeneity: hyphal growth unit

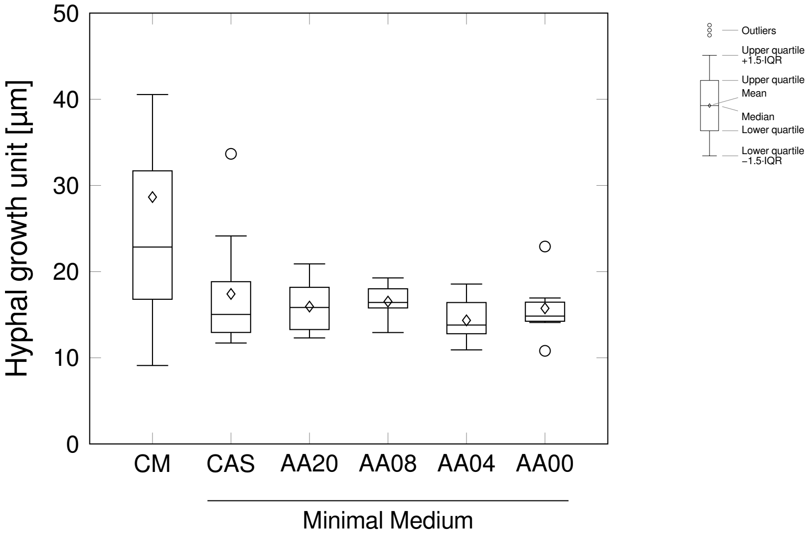

Figure S5. Hyphal growth unit, defined as number of branches per total mycelium length. Box plot values are provided for complex (CM) and different minimal medium conditions (CAS, AA20 – AA00). Box plot annotations as indicated.
