# Supplementary material for: Germination and Growth Analysis of Streptomyces lividans at the Single-Cell Level Under Varying Medium Compositions
Source: Front Microbiol. 2018 Nov 22;9:2680. doi: 10.3389/fmicb.2018.02680 (PMC6262040; doi:10.3389/fmicb.2018.02680)
Supplement: Supplementary file 2 [file Data_Sheet_2.ZIP › Raw Analysed Data/Streptomycete_Analysis.html]

Streptomycete\_Analysis


# “Germination and Growth Analysis of Streptomyces lividans at the Single-Cell Level Under Varying Medium Compositions”¶

by Joachim Koepff, Christian Carsten Sachs, Wolfgang Wiechert, Dietrich Kohlheyer, Katharina Nöh, Marco Oldiges and Alexander Grünberger

## Analyses¶

The following cell contains the filename of the HDF5 (`.h5`) to load, as well as a hash to check its integrity.

Note, that within the analyses here 'CM' (Complex Medium) is just called 'Complex'.

In [1]:

```
file_name = 'Streptomycete_Results_Compact.h5'
hash_sha256 = '405e6a9bb270eb2518115b92f75301a7d6deaeeb65460045861f5eb6cb7272b6'
order = 'Complex, CAS, AA20, AA08, AA04, AA00'
```

First, the necessary packages are loaded, this notebook has been tested with Python 3.6.6, numpy 1.15.1, scipy 1.1.0, matplotlib 2.2.2 and pandas 0.23.4.

In [2]:

```
%matplotlib inline
%config InlineBackend.figure_format = 'svg'
import numpy as np
from matplotlib import pyplot
import pandas
import hashlib
try:
    import seaborn
    seaborn.set()
except ImportError:
    print("You could install seaborn to have prettier plots, but it is optional.")
from scipy.ndimage import gaussian_filter1d
```

In [3]:

```
if isinstance(order, type("")):
    order = order.split(', ')

current_hash_sha256 = hashlib.sha256(open(file_name, 'rb').read()).hexdigest()

if hash_sha256 != current_hash_sha256:
    print("WARNING: HDF5 file does not match original checksum! Results may be different.")
    # Comment out the next line if you want to use this script with other data
    assert False
    
hour_to_second = per_second_to_per_hour = 60.0 * 60.0
per_hour_to_per_second = second_to_hour = 1.0 / per_second_to_per_hour
data_store = pandas.HDFStore(file_name, 'r')
```

A set of helper functions to access the data are defined.

In [4]:

```
def recursive_list_nodes(hdf5, start=(), depth=0):
    memo = []

    def _inner(node, cur_depth):
        if cur_depth == 0:
            return
        for n in hdf5.list_nodes(where='/' + '/'.join(node)):
            new_node = node + (n._v_name,)
            memo.append(new_node)
            _inner(new_node, cur_depth - 1)

    _inner(start, depth)
    return memo


def merge(data_store, contents, ending_with):
    return pandas.concat([data_store['/' + '/'.join(item)] for item in contents if item[-1] == ending_with])


def build_track_table(data_store, contents):
    tables = []
    for item in contents:
        if item[-1] != 'result_table':
            continue
        different_path = item[:-1] + ('tables', 'track_table', 'track_table_000000000',)
        try:
            _result_table = data_store['/' + '/'.join(item)]
            _track_table = data_store['/' + '/'.join(different_path)]

            _track_table['path'] = ['/' + '/'.join(different_path)] * len(_track_table)
            _track_table['metadata'] = [str(_result_table.metadata[0])] * len(_track_table)
            _track_table['meta_pos'] = [int(_result_table.meta_pos[0])] * len(_track_table)
            _track_table['filename'] = [str(_result_table.filename[0])] * len(_track_table)
            _track_table['filename_complete'] = [str(_result_table.filename_complete[0])] * len(_track_table)

            tables.append(_track_table)
        except KeyError as e:
            print(">>> ", e)
    return pandas.concat(tables)

def get_track_by_trackrow(row):
    
    path_split = row.path.split('/')
    path_split = path_split[:-2]
    
    mapping = '/'.join(path_split + ['_mapping_track_table_aux_tables/track_table_aux_tables_000000000'])
    
    aux_table_idx = int(data_store[mapping].query('_index == @row.aux_table').individual_table)
    
    realpath = '/'.join(path_split + ['_individual_track_table_aux_tables/track_table_aux_tables_%09d' % aux_table_idx])
    
    data = data_store[realpath]
    return data

def first(what):
    return next(iter(what))
```

… and the results are loaded

In [5]:

```
contents = recursive_list_nodes(data_store._handle, depth=4)
result_table = merge(data_store, contents, 'result_table')
result_table_collected = merge(data_store, contents, 'result_table_collected')
track_table_merged = build_track_table(data_store, contents)
```

# Figure 2B¶

In [6]:

```
def figure2B():
    metadata = 'Complex'
    subset = result_table_collected.query('metadata == @metadata')

    to_output = []

    fig = pyplot.figure()
    ax = fig.add_subplot(1, 1, 1)

    for (filename, meta_pos), grouped in subset.groupby(by=['filename', 'meta_pos']):
        info = result_table.query('metadata == @metadata and filename == @filename and meta_pos == @meta_pos')

        slope = first(info.graph_edge_length_optimized_logarithmic_regression_slope)
        if slope != slope:
            continue

        opt_end = first(info.graph_edge_length_optimized_logarithmic_regression_end) * second_to_hour
        opt_beg = first(info.graph_edge_length_optimized_logarithmic_regression_begin) * second_to_hour

        lengths = np.array(grouped.graph_edge_length)
        times = np.array(grouped.timepoint) * second_to_hour

        # subtract minimum: only we are only interested in additional length 
        lengths -= lengths.min()

        # as artifacts are within the data,
        # filter to only the exponential growth range
        lengths = lengths[times < opt_end]
        times = times[times < opt_end]

        # max filter: trim time series to up to maximum length value
        max_pos = np.argmax(lengths)
        times = times[:max_pos]
        lengths = lengths[:max_pos]

        # during early stages of the analysis, convolution with a Hamming window was used
        # smoothing using Gaussian sigma 1.346 roughly equates to smoothing using a Hamming window n=5
        sigma = 1.34649727
        gl = np.diff(gaussian_filter1d(lengths, sigma))

        gl[gl > 0] = 0
        threshold = -50
        gl[gl > threshold] = 0
        gl[gl < threshold] = 1
        premature, = np.where(gl)
        
        # some artifacts present as steep length reductions near the end, clip them out

        if len(premature) > 0:
            premature = premature[0]

            if premature < len(lengths) / 2:
                times = times[premature:]
                lengths = lengths[premature:]
            else:
                times = times[:premature]
                lengths = lengths[:premature]

        to_output.append((times, lengths))
        ax.plot(times, lengths)

    ax.set_title("Figure 2B – Mycelia length over time")
    ax.set_xlim(0, 24)
    ax.set_xlabel("Time [h]")
    ax.set_ylabel("Mycelium length [µm]")


figure2B()
```

xml version="1.0" encoding="utf-8" standalone="no"?

# Figure 2D¶

In [7]:

```
def figure2D():
    the_result = {}
    
    prefix='graph_edge_length_optimized_logarithmic_regression'
    
    # optimized regression longer than 5h
    minimum_regression_length = 5.0
    filter_string = '(%s_end - %s_begin) > %.2f' % (prefix, prefix, minimum_regression_length * hour_to_second)
    
    for metadata, grouped in result_table.groupby(by=['metadata']):
        lengths = grouped.query(filter_string)["%s_slope" % (prefix,)]
        
        lengths = np.array(lengths)
        lengths *= per_second_to_per_hour
        
        the_result[metadata] = lengths[np.isfinite(lengths)]
    
    fig = pyplot.figure()
    ax = fig.add_subplot(1, 1, 1)
    
    bp = ax.boxplot([the_result[m] for m in order], labels=order, positions=np.arange(0, len(order)*2, 2), showmeans=True)
    
    ax.set_title("Figure 2D – Growth rate")
    
    ax.set_xticks(np.arange(0, len(order)*2, 2))
    ax.set_xlim(-2, len(order) * 2)
    ax.set_ylim(0.0, 0.7)
    ax.set_ylabel("Growth rate µ [h$^{-}$¹]")
    
    ax.legend([bp['boxes'][0]], ["by length"])
        
figure2D()
```

xml version="1.0" encoding="utf-8" standalone="no"?

# Figure 3B¶

In [8]:

```
def figure3B():
    the_result = {}
    
    for (metadata, filename, meta_pos), grouped in track_table_merged.groupby(by=['metadata', 'filename', 'meta_pos']): 
        if metadata not in the_result:
            the_result[metadata] = []
            
        grouped = grouped.copy()
        grouped = grouped.sort_values(by='timepoint_begin')
        
        time_deltas = np.array(grouped['timepoint_end'] - grouped['timepoint_begin'])[0]
        
        
        track_starts = np.array(grouped['timepoint_begin'])[0]
        
        track_starts = track_starts * second_to_hour
        
        the_result[metadata].append(track_starts)

    fig = pyplot.figure()
    ax = fig.add_subplot(1, 1, 1)
    ax.set_title("Figure 3B – Germination delay")
        
    bp = ax.boxplot([the_result[m] for m in order], labels=order, positions=np.arange(0, len(order)*2, 2), showmeans=True)
    
    ax.set_xticks(np.arange(0, len(order)*2, 2))
    ax.set_xlim(-2, len(order) * 2)
    ax.set_ylabel("Germination delay [h]")
    ax.set_ylim(0, 40)
    ax.legend([bp['boxes'][0]], ["by length"])

figure3B()
```

xml version="1.0" encoding="utf-8" standalone="no"?

# Figure 4A¶

In [9]:

```
def figure4A():
    filter_string = 'metadata == "Complex" and meta_pos == 138'

    _, subset = first(track_table_merged.query(filter_string).groupby(by=['metadata', 'filename', 'meta_pos']))

    fig = pyplot.figure()
    ax = fig.add_subplot(1, 1, 1)
    ax.set_title("Figure 4A – Hyphae elongations")

    for _, row in subset.iterrows():
        d = get_track_by_trackrow(row)

        times = np.array(d.timepoint)
        times = times * second_to_hour

        lengths = np.array(d.distance)
        lengths -= lengths.min()

        ax.plot(times, lengths)

    ax.set_xlim(0, 24)
    ax.set_xlabel("Time [h]")
    ax.set_ylim(0, 100)
    ax.set_ylabel("Hyphae elongation [µm]")

figure4A()
```

xml version="1.0" encoding="utf-8" standalone="no"?

# Figure 4B¶

In [10]:

```
def figure4B():
    filter_string = 'metadata == "Complex"'

    fig = pyplot.figure()
    ax = fig.add_subplot(1, 1, 1)
    ax.set_title("Figure 4B – Tip count over time")

    for (filename, meta_pos), subset in result_table_collected.query(filter_string).groupby(
            by=['filename', 'meta_pos']):

        rt = result_table.query('filename == @filename and meta_pos == @meta_pos')
        tt = track_table_merged.query('filename == @filename and meta_pos == @meta_pos')

        slope = first(rt.graph_edge_length_optimized_logarithmic_regression_slope)

        # skip nan slopes
        if slope != slope:
            continue

        begin, end = tt.timepoint_begin.min(), tt.timepoint_end.max()

        tips = np.array(subset.graph_endpoint_count)
        times = np.array(subset.timepoint)

        idx = (times > begin) & (times < end)

        tips = tips[idx]
        times = times[idx]

        times *= second_to_hour

        times_filtered, tips_filtered = [], []
        last_tip_count = -1

        for time, tip_count in zip(times, tips):
            if tip_count > last_tip_count:
                last_tip_count = tip_count
            tips_filtered.append(last_tip_count)
            times_filtered.append(time)

        # remove artifacts
            
        last_tip_count = tips_filtered[-1]

        j = len(tips_filtered) - 1

        while j > 0:
            j -= 1
            
            if tips_filtered[j] != last_tip_count:
                break

        j += 2

        if (len(tips_filtered) - j) > 5:
            times_filtered = times_filtered[:j]
            tips_filtered = tips_filtered[:j]

        if len(tips_filtered) == 0:
            continue

        if max(tips_filtered) <= 5:
            continue

        ax.plot(times_filtered, tips_filtered)

    ax.set_xlabel('Time [h]')
    ax.set_xlim(0, 25)
    ax.set_ylabel('Hyphae Tips [#]')

figure4B()
```

xml version="1.0" encoding="utf-8" standalone="no"?

Some shared helper code for Figure 4 C, D and S4

In [11]:

```
def helper_figure4CD_S4(metadata):
    result_data = {}

    subset_outer = track_table_merged.query('metadata == @metadata')

    for (filename, meta_pos), subset in subset_outer.groupby(by=['filename', 'meta_pos']):
        arr = []
        for n, row in subset.sort_values(by=['timepoint_begin']).iterrows():
            if row.plain_regression_rvalue == 1.0:
                continue

            slope = row.plain_regression_slope
            # slope = row.optimized_regression_slope

            if slope != slope:
                continue

            slope = slope * per_second_to_per_hour
            arr.append(slope)

        if len(arr) < 5:
            continue

        result_data[(metadata, filename, meta_pos)] = np.array(arr)

    return result_data


def helper_figure4C_S4_plotter(ax, metadata):
    ax.set_title("%s" % (metadata,))

    array_of_arrays = list(helper_figure4CD_S4(metadata).values())
    array_of_arrays = sorted(array_of_arrays, key=np.mean)

    titles = ["S%d" % (n + 1,) for n in range(len(array_of_arrays))]

    titles = [metadata] + list(titles)
    array_of_arrays = [np.concatenate(list(array_of_arrays))] + list(array_of_arrays)

    ax.boxplot(array_of_arrays, labels=titles, showmeans=True)
    ax.set_ylim(0, 50)
    ax.set_ylabel("Tip elongation rate [µm·h$^{-}$¹]")
```

# Figure 4C¶

In [12]:

```
def figure4C():
    fig = pyplot.figure()
    ax = fig.add_subplot(1, 1, 1)
    helper_figure4C_S4_plotter(ax, 'Complex')
    
    ax.set_title("Figure 4C – Tip elongation rates for Complex medium")

figure4C()
```

xml version="1.0" encoding="utf-8" standalone="no"?

# Figure 4D¶

In [13]:

```
def figure4D():
    fig = pyplot.figure()
    ax = fig.add_subplot(1, 1, 1)
    
    ax.set_title("Figure 4D – Mean tip elongation rates")

    array_of_values = [
        [np.mean(values) for values in helper_figure4CD_S4(metadata).values()]
        for metadata in order
    ]
    
    ax.bar(order, list(map(np.mean, array_of_values)), yerr=list(map(np.std, array_of_values)))
    
    ax.set_ylabel("Mean tip elongation rate [µm·h$^{-}$¹]")


figure4D()
```

xml version="1.0" encoding="utf-8" standalone="no"?

# Figure S4¶

In [14]:

```
def figureS4():
    height = 4
    stretch = 1.5
    fig, axarr = pyplot.subplots(len(order)//2, 2, sharey=False, figsize=(2 * height * stretch, len(order)//2 * height))

    fig.suptitle("Figure S4 – Tip elongation rates for different media")

    for ax, metadata in zip(axarr.flat, order):
        helper_figure4C_S4_plotter(ax, metadata)

figureS4()
```

xml version="1.0" encoding="utf-8" standalone="no"?

# Figure S5¶

In [15]:

```
def figureS5():
    result_data = {m: [] for m in order}

    for (filename, meta_pos), subset in result_table_collected.groupby(by=['filename', 'meta_pos']):
        metadata = list(subset.metadata)[0]

        rt = result_table.query('filename == @filename and meta_pos == @meta_pos')

        slope = first(rt.graph_edge_length_optimized_logarithmic_regression_slope)
        if slope != slope:
            continue

        begin, end = first(rt.graph_edge_length_optimized_logarithmic_regression_begin), first(rt.graph_edge_length_optimized_logarithmic_regression_end)

        lengths = np.array(subset.graph_edge_length)
        tips = np.array(subset.graph_endpoint_count)
        
        times = np.array(subset.timepoint)

        idx = (times > begin) & (times < end)

        lengths = lengths[idx]
        tips = tips[idx]
        # times = times[idx]

        # during early stages of the analysis, convolution with a Hamming window was used
        # smoothing using Gaussian sigma 1.959 roughly equates to smoothing using a Hamming window n=10
        sigma = 1.95979286
        
        # smooth the values by convolving with a Gaussian kernel as to reduce impact of artifact values

        # times = gaussian_filter1d(times, sigma)
        lengths = gaussian_filter1d(lengths, sigma)

        with np.errstate(divide='ignore', invalid='ignore'):
            hgu = lengths / tips

        max_val = np.max(hgu)

        result_data[metadata].append(max_val)

    fig = pyplot.figure()
    ax = fig.add_subplot(1, 1, 1)
    
    ax.set_title("Figure S5 – Hyphal growth unit")

    ax.boxplot([result_data[m] for m in order], labels=order, positions=np.arange(0, len(order)*2, 2), showmeans=True)

    ax.set_xticks(np.arange(0, len(order)*2, 2))
    ax.set_xlim(-2, len(order) * 2)
    ax.set_ylim(0, 50)
    ax.set_ylabel('Hyphal growth unit [µm]')

figureS5()
```

xml version="1.0" encoding="utf-8" standalone="no"?
